# A Novel Role of Bergamottin in Attenuating Cancer Associated Cachexia by Diverse Molecular Mechanisms

**DOI:** 10.3390/cancers13061347

**Published:** 2021-03-17

**Authors:** Young Yun Jung, Jeong-Hyeon Ko, Jae-Young Um, Gautam Sethi, Kwang Seok Ahn

**Affiliations:** 1 Department of Science in Korean Medicine, Kyung Hee University, 24 Kyungheedae-ro, Dongdaemun-gu, Seoul 02447, Korea; ve449@naver.com (Y.Y.J.); gokjh1647@gmail.com (J.-H.K.); jyum@khu.ac.kr (J.-Y.U.); 2 Department of Pharmacology, Yong Loo Lin School of Medicine, National University of Singapore, Singapore 117600, Singapore

**Keywords:** cachexia, bergamottin, STAT3, ERK, conditioned media

## Abstract

**Simple Summary:**

Cachexia has been generally associated with cancer causing skeletal muscle atrophy, adipose tissue atrophy, weight loss, anorexia, asthenia, and anemia, which can significantly reduce the quality of life. Our aim was to evaluate the potential effects of bergamottin on cancer-cachexia-induced muscle and fat loss. We observed a decrease in the levels of the muscle atrophy factors MuRF-1 and Atrogin-1 and increases in C/EBPα and PPARγ expression levels by bergamottin under in vitro settings. The in vivo effect of bergamottin on the inhibition of weight loss in mice and its potential inhibitory effects on cancer-induced cachexia were confirmed through analysis using tissue samples from a pancreatic cancer mouse model.

**Abstract:**

Purpose: The potential effects of bergamotiin (BGM) on the suppression of cancer cachexia was evaluated under in vitro and in vivo conditions to investigate its possible inhibitory effects on the muscle and fat loss. Method: The differentiated C2C12 and 3T3L1 cells were treated with BGM after the induction of cancer-cachexia with pancreatic cancer conditioned media (CM). The expression levels of the various molecules involved in the differentiation and loss of muscle and fat (MuRF-1, Atrogin-1, C/EBPα, and PPARγ) were analyzed by Western blot and oil red O staining. For in vivo experiment, MIA PaCa-2 cells were injected into the mice (*n* = 6), and then BGM (1 mg/kg) was intraperitoneally administered to analyze muscle and adipose tissue by Hematoxylin and Eosin staining and Western blot. Result: BGM displayed a significant effect on the inhibition of muscle and fat catabolism under both in vitro and in vivo conditions. The results of the in vivo experiment revealed a remarkable suppressive effect of BGM on the weight loss in mice. Conclusions: The potential effects of BGM on the inhibition of muscle and fat catabolism in vitro and in vivo were thus confirmed. Based on the results, the impact of BGM on cancer cachexia could be possibly analyzed in the future clinical studies.

## 1. Introduction

Cachexia is a major multifactorial syndrome, which can affect patients with cancer. Cachexia can reduce the patient’s resistance to cancer and approximately could account for skeletal muscle atrophy, adipose tissue atrophy, weight loss, anorexia, asthenia, and anemia that can lead to degradation of quality of life in patients, reduce their responsiveness to chemotherapy and thereby cause poor prognosis [1,2,3,4]. These processes are generally considered as the major features of malignant disease, which can lead to death. Cachexia can also alter important energy sources like skeletal muscles and adipose tissues, causes asthenia and can lead to lack of nutrition [5]. Cachexia can reduce patient’s resistance to cancer and approximately can account for about 20% of cancer-related mortality worldwide [6].

Muscle atrophy is generally caused by an unbalance in anabolic and catabolic processes, when protein breakdown can exceed protein synthesis processes [7]. It can lead to a significant muscle loss, and in 1969 Goldberg elegantly demonstrated that increased protein breakdown can substantially contribute to muscle mass and myofibrillar proteins loss [7,8]. It has been reported that muscle loss can be enhanced during cancer, sepsis, starvation, and diabetes, but the proteolysis signaling pathway regulating this process was not well characterized [9]. In 2001, however, reports suggested that E3 ubiquitin ligases can function as critical regulators of muscle atrophy [10,11]. In a study by Bodine et al. [10], *MuRF-1* (Trim63) and *MAFbx* (FBX032) genes were identified to be involved in this process by a differential display approach. For instance, MuRF-1 showed a significant increase in resting skeleton, whereas Atrogin-1 was found to be enhanced in different atrophy models [11,12]. Atrogin-1 showed a remarkable increase in both diabetic and cancer cachexia models, thereby indicating that the *MuRF-1* gene level can increase during the protein catabolic phenomena [11,13].

Cachexia is also characterized by significant fat mass loss, and it has been found to be associated with adipocyte and adipose tissue atrophy. Atrophy can be activated in adipocytes, which can reduce the volume of cells and also decrease de novo lipogenesis [14,15,16,17]. Adipose tissue has been found to regulate tumor microenvironment and inflammation by secreting several hormones such as adiponectin and leptin [18]. In our body, fat droplets in white adipose tissue (WAT) can store energy and produce inflammatory mediators such as adiponectin, leptin, IL-6, IL-1β, TNF-α, and zinc-a2 glycoprotein (ZAG). These molecules enable adipocytes to respond to external stimuli through unique metabolism and can participate in various physiological processes [17,18,19]. C/EBPα and PPARγ have been reported to be involved in adipogenesis regulation and can control adipocyte differentiation [20]. Moreover, adipokine genes *aP2*, adiponectin, and resistin can also modulate adipocyte-specific gene expression [20,21]. However, cancer cachexia can promote adipose- and inflammation-associated disorders, causing an increase in the levels of systemic proinflammatory factors [3]. Furthermore, dysfunctional adipose-derived stem cells and cancer-associated adipocytes can also contribute to the process of tumorigenesis [22].

Our research is primarily focused on the prevention and treatment of various chronic diseases including cancers through the application of naturally derived products that have been reported to display significant efficacy against various neoplasms [23,24,25,26,27,28]. Furanocoumarins are a kind of natural pesticides that can regulate defense mechanisms in plants [29,30]. One important component bergamottin (BGM) belonging to this class is commonly found in various citrus fruits including the pulp of grapefruits and pomelos [31,32,33]. The possible effects of BGM on weight regulation and adipocyte differentiation has been reported previously [34,35]. Additionally, the studies about multimodal interventions for the management of cachexia in cancer have been reported [36,37]. Hence, in this study, the potential impact of BGM on the regulation of cancer cachexia and its action on muscle and adipose tissue atrophy has been deciphered. The findings suggest that BGM can attenuate both cancer-associated cachexia and tumorigenesis in a pancreatic cancer model.

## 2. Results

### 2.1. BGM Suppresses the Pancreatic Cancer Conditioned Media (CM)-Induced Cancer Cachexia in C2C12 Mouse Myoblast Cells In Vitro

**Figure 1:**
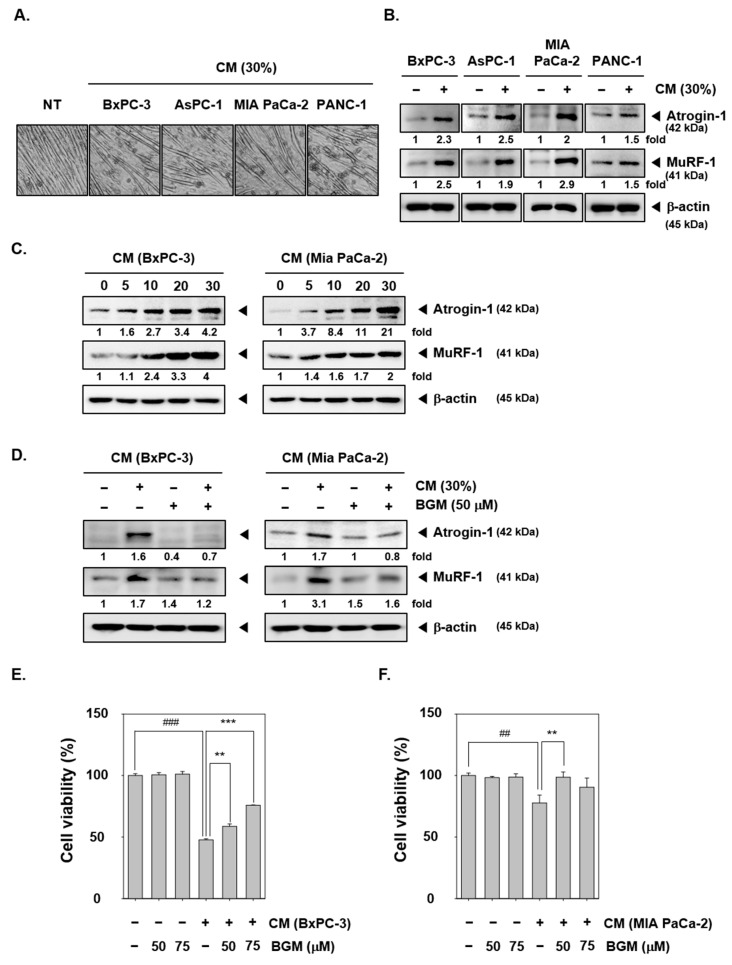
Suppressive effects of bergamotiin (BGM) upon cancer cachexia-induced muscle cells atrophy. C2C12 cells were stimulated with CT26 cells conditioned media (CM) for 3 days. (**A**) C2C12 cells induced cancer cachexia by incubation with various cancer cells CM for 24 h and morphological changes were observed. (**B**) C2C12 cells were incubated with 30% CM for 24 h, which were obtained from BxPC-3, AsPC-1, MIA PaCa-2, and PANC-1 cells. Then, the expression levels of E3 ubiquitin ligases Atrogin-1 and MuRF-1 were observed by Western blot analysis. (**C**) C2C12 cells were incubated with various concentrations of CM and evaluated by Western blot analysis. (**D**) C2C12 cells were treated by BGM (50 µM) with BxPc-3 and MIA PaCa-2 cells 30% CM for 24 h. Thereafter, Atrogin-1 and MuRF-1 expression levels were analyzed by Western bolt analysis. (**E**,**F**) C2C12 cells were treated with BGM (0, 50, and 75 µM) with BxPc-3 and MIA PaCa-2 cells 30% CM for 48 h. Then, the cell viability was measured by the 3-(4,5-dimethylthiazol-2-yl)-2,5-diphenyltetrazolium bromide (MTT) assay. All the experiments were performed independently at least 3 times, and representative data are shown. Data represent means ± SD. ## *p* < 0.01 vs. NT, ### *p* < 0.001 vs. NT, ** *p* < 0.01 vs. BGM, *** *p* < 0.001 vs. BGM.

To promote the differentiation of C2C12 myoblast cells, we seeded these cells with high density and changed the media with 2% horse serum for three days regularly. Then, cancer cachexia was induced with 30% CM (diluted by serum-free DMEM media) from various pancreatic cancer cells, such as BxPC-3, AsPC-1, MIA PaCa-2, and PANC-1 cells [38]. As shown in Figure 1A, the morphology of cells was first observed, and cachexia was confirmed upon exposure to 30% pancreatic cancer CM conditions. In non-treated (NT) C2C12 cells, the differentiation into myotubes resulted into a thick and elongated structures. However, the structure of CM-treated cells became thinner, which indicated a possible progressive degradation. Thereafter, Atrogin-1 and MuRF-1 expression was evaluated by Western blot analysis (Figure 1B). C2C12 cells were incubated with the various concentrations of BxPC-3 and MIA PaCa-2 CM to evaluate the protein expression levels of Atrogin-1 and MuRF-1 (Figure 1C). Atrogin-1 and MuRF-1 are muscle-specific E3 ubiquitin ligases, which are involved in the regulation of muscle atrophy in cancer cachexia. As we can observe myotube atrophy by CM in Figure 1A, Atrogia-1 and MuRF-1 expression levels were induced under the similar conditions. Thus, the progression of cancer cachexia as clearly demonstrated can be related to the expression of Atrogin-1 and MuRF-1. We have selected BxPC-3 and MIA PaCa-2 CM as cancer cachexia inducers in C2C12 myotubes because of their significant observed effects. We next investigated the suppression effects of BGM on Atrogin-1 and MuRF-1 expression by Western blot analysis (Figure 1D). As shown, when BxPC-3 or MIA PaCa-2 CM was added, Atrogin-1 and MuRF-1 expression levels were strongly induced. However, the BGM-treated cells failed to induce both the ligases and also could not restore CM-induced ligase expression. Thereafter, the cell viability was measured after the treatment with CM and BGM. The BxPC-3- and MIA PaCa-2 CM-treated C2C12 myotubes showed a significant decrease in the cell viability, but interestingly BGM can suppress cancer cachexia-induced cell death (Figure 1E,F). These results indicated that BGM can effectively decrease cell death through the suppression of cancer cachexia.

### 2.2. BGM Can Induce the Myosin Heavy Chain (MyHC) Expression Cancer Cachexia-Induced C2C12 Myotubes In Vitro

**Figure 2:**
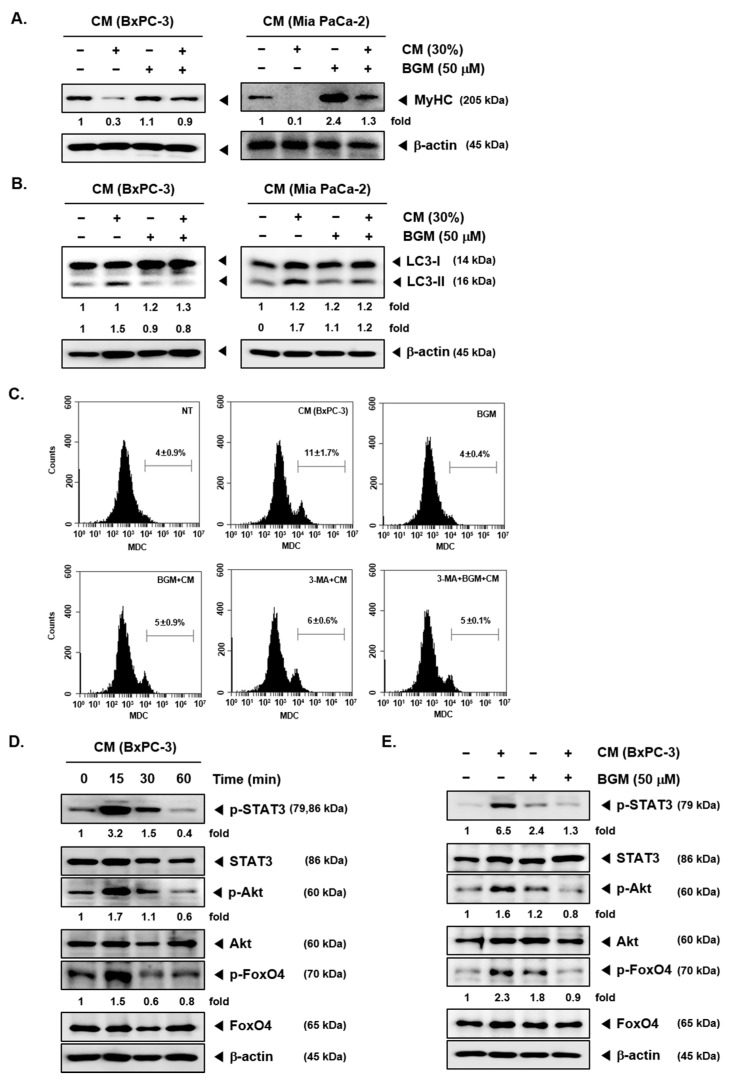
Effects of BGM on autophagy activation and various cancer markers suppression levels in C2C12 cells. C2C12 cells were treated by BGM (50 µM) with 30% of BxPc-3 and MIA PaCa-2 cells CM for 24 h. The lysates were analyzed by Western blot analysis and probed with myosin heavy chain (MyHC; **A**) and light chain 3 (LC3; **B**) antibodies. (**C**) C2C12 cells were treated by BGM (50 µM) with BxPC-3 CM for 24 h and then incubated with the autophagy inhibitor 3-methyladenine (3-MA). Thereafter, the cells were stained with monodansylcadaverine (MDC) and analyzed by flow cytometry. (**D**) C2C12 cells were incubated with 30% BxPC-3 CM at various time intervals to select the optimal expression time for various cancer markers. Then, 15 min of BxPC-3 CM stimulation could effectively induce the expression of cancer markers. (**E**) C2C12 cells were treated with BGM (50 µM) and then stimulated for 15 min with BxPC-3 CM. The lysates were analyzed by Western blot analysis for various proteins. All the experiments were performed independently at least 3 times, and representative data are shown.

MyHC is one of important skeletal muscle gene products [39]. We evaluated the ability of BGM to restore the MyHC expression, because MyHC expression has been reported to be attenuated under conditions when cancer cachexia is induced (Figure 2A). When BxPC-3 and MIA PaCa-2 CM were added to C2C12 myotubes, MyHC expression was clearly suppressed as compared to NT C2C12 cells. However, BGM-treated C2C12 cells were able to recover their MyHC at levels similar to NT C2C12 cells. These findings suggested that BGM can also modulate skeletal muscle gene products expression in cancer cachexia-induced C2C12 myotubes.

### 2.3. Cancer Cachexia-Induced Autophagy Can Be Suppressed by BGM in C2C12 Myotubes In Vitro

We next analyzed the autophagy activation in CM-treated C2C12 myotubes with microtubule-associated protein 1A/1B-light chain 3 (LC3) expression by Western blot analysis. As shown by the results, LC3-II expression was increased by both BxPC-3 and MIA PaCa-2 CM. However, BGM exposure leads to a substantial decrease in CM-induced LC3-II increase (Figure 2B). Thereafter, the autophagy suppression by BGM was confirmed by monodansylcadaverine (MDC)-stained cell sorting using a flow cytometer (Figure 2C). It was observed that MDC can selectively stain acidic components, which are established representative feature of autophagy. We analyzed BxPC-3 CM-treated cells for this experiment, as this cell line showed more marked results by Western blot analysis. The data clearly indicated that BxPC-3 CM-induced autophagy as observed with MDC-stained cells; however, BGM suppressed MDC staining, thus causing autophagy inhibition. The number of stained cells indicating autophagy can be clearly established through the dramatic reduction in the number of MDC-stained cells upon treatment with the autophagy inhibitor 3-methyladenine (3-MA).

### 2.4. BGM Inhibits Cachexia-Induced Inflammatory Signaling Factors in C2C12 Myotubes In Vitro

To evaluate the process of cancer development in BxPC-3 CM-treated C2C12 cells, we analyzed STAT3, Akt, and FoxO4 phosphorylation by Western blot analysis. We first exposed C2C12 cells to CM at various time intervals to select the optimum time point and found that 15 min incubation was the best condition for STAT3, Akt, and FoxO4 maximal activation (Figure 2D). However, BGM could effectively downregulate the STAT3, Akt, and FoxO4 phosphorylation despite the occurrence of CM-induced cachexia (Figure 2E). The results demonstrated that BGM can also exert significant antineoplastic effects upon C2C12 myotubes.

### 2.5. Cancer Cachexia Is Induced by CM in 3T3L1 Mouse Fibroblast Cells In Vitro

**Figure 3:**
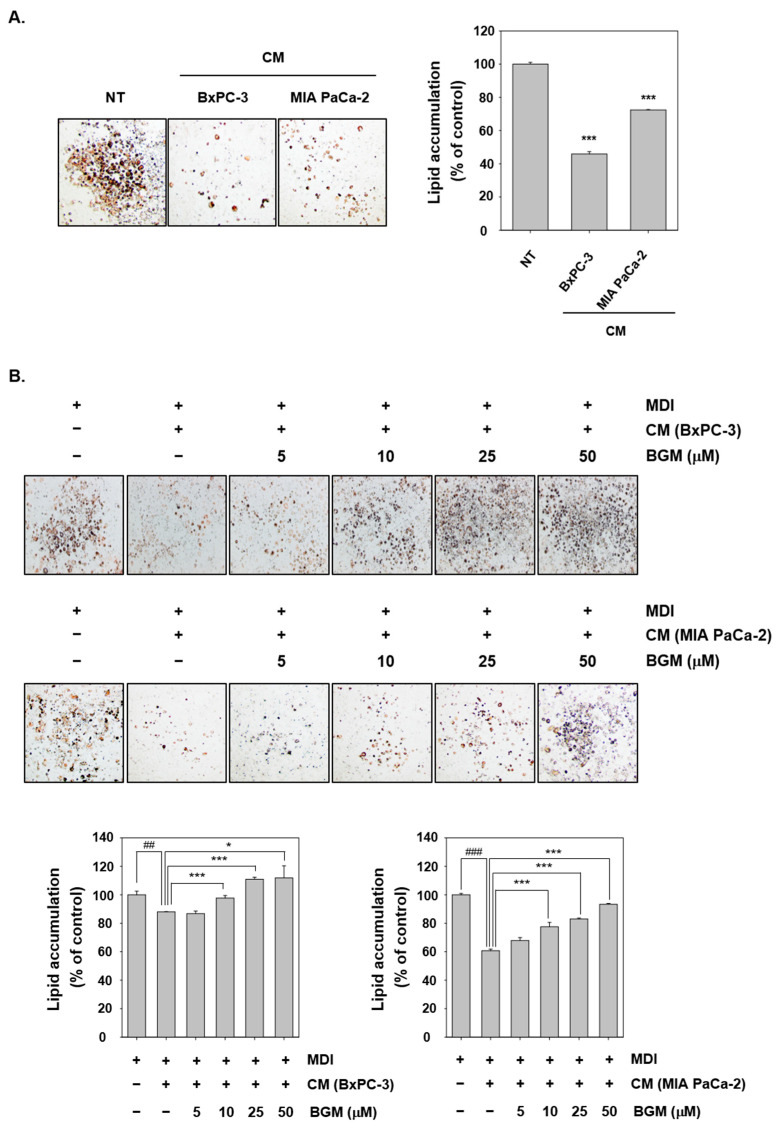
Suppressive actions of BGM on cancer cachexia could induce atrophy of adipocytes. (**A**) 3T3-L1 cells were incubated with MDI media for 3 days to cause differentiation into adipocytes and thereafter stimulated with BxPC-3 and MIA PaCa-2 CM for 2 days. The lipid droplets were observed by oil red staining, and lipid accumulation was measured by VARIOSKAN LUX (Thermo Fisher Scientific Inc, Waltham, MA, USA). (**B**) 3T3-L1 cells were differentiated into adipocytes and then treated with BGM (0, 5, 10, 25, and 50 µM) along with BxPC-3 and MIA PaCa-2 CM for 2 days. Adipogenesis was observed by oil red staining and measured by VARIOSKAN LUX (Thermo Fisher Scientific Inc., Waltham, MA, USA). All the experiments were performed independently at least 3 times, and representative data are shown. Data represent means ± SD. ## *p* < 0.01 vs. NT, ### *p* < 0.001 vs. NT, * *p* < 0.05 vs. BGM, *** *p* < 0.001 vs. BGM.

To investigate the cancer cachexia in 3T3L1 mouse fibroblast cells, cell differentiations into adipocyte were induced by MDI (500 µM IBMX, 1 µM Dexamethasone, and 1 µg/mL Insulin) media for three days. Thereafter, the cells were replaced with media or CM, including insulin and BGM for two days. Finally, all the cells were incubated with insulin containing media for four days. We first confirmed that both BxPC-3 and MIA PaCa-2 CM can induce adipocytes atrophy by oil red O staining (Figure 3A). Because oil red O could effectively stain neutral lipids in hepatocytes, they can potentially be used as a marker of adipocytes differentiation. We noted that as compared with CM NT cells, only few cells were stained by oil red O after both BxPC-3 and MIA PaCa-2 CM treatment. Then, the cells were lysed with ethanol, and their lipid accumulation was measured by VARIOSKAN LUX. Thereafter, the effects of BGM on adipocytes atrophy was evaluated. Adipocytes were treated with BGM at various concentrations and analyzed by oil red O staining (Figure 3B). In both BxPC-3- and MIA PaCa-2 CM-induced cells, the percentage of oil red O stained cells increased dramatically with increasing BGM concentration. The lipid accumulation was measured by VARIOSKAN LUX, and the results clearly indicated that BGM could inhibit the cancer cachexia process through adipocytes atrophy suppression in 3T3L1 cells.

### 2.6. BGM Can Induce Adipogenesis- and Differentiation-Related Factors in 3T3L1 Cells In Vitro

**Figure 4:**
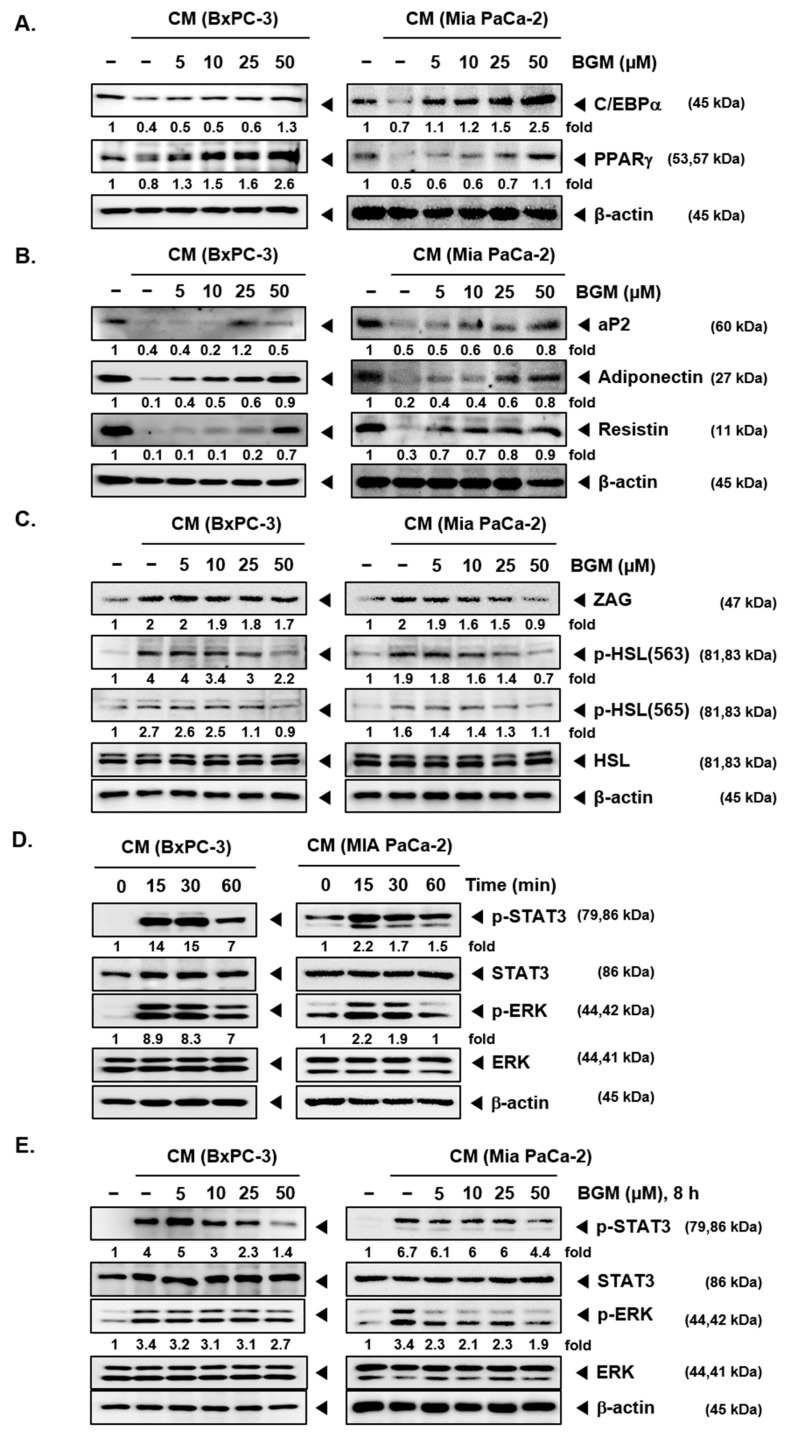
Induction of adipogenesis markers in differentiated 3T3-L1 cells by BGM. (**A**) 3T3-L1 cells were differentiated into adipocytes and then treated with BGM in a dose-dependent manner along with BxPC-3 and MIA PaCa-2 CM for 2 days. The expression levels of adipogenesis-related factors C/EBPα and PPARγ were analyzed by Western blot analysis. (**B**) The levels of adipocytes markers *aP2*, adiponectin, and resistin were analyzed by Western blot analysis. (**C**) 3T3-L1 cells were incubated with BxPC-3 and MIA PaCa-2 CM for 8 h. The expression levels of zinc-a2 glycoprotein (ZAG), p-hormone-sensitive lipase (HSL), and HSL were evaluated by Western blot analysis. (**D**) 3T3-L1 cells were incubated with BxPC-3 and MIA PaCa-2 CM in a time-dependent fashion. (**E**) The differentiated 3T3-L1 cells were treated with BGM (0, 5, 10, 25, and 50 µM) for 8 h and then incubated with BxPC-3 and MIA PaCa-2 CM for 15min. The expression levels of p-STAT3, STAT3, p-ERK, and ERK were observed by Western blot analysis. All the experiments were performed independently at least 3 times, and representative data are shown.

C/EBPα and PPARγ have been known as adipogenesis regulation factors through adipocyte differentiation. To evaluate the BGM effects on adipogenesis, we analyzed C/EBPα and PPARγ by Western blot analysis (Figure 4A). Although CM suppressed the C/EBPα and PPARγ expression, BGM can clearly upregulate the protein levels of both factors. Then, we confirmed the BGM-induced adipogenic marker and the adipokine genes *aP2*, adiponectin, and resistin by Western blot analysis (Figure 4B). As shown by the results, *aP2*, adiponectin, and resistin expression levels were increased with BGM treatment, showing that BGM inhibits cachexia-induced adipocytes atrophy by inducing the expression of adipogenesis genes and factors. ZAG and hormone-sensitive lipase (HSL) have been reported to stimulate adipocyte lipid metabolism. ZAG and HSL can also induce lipid catabolism in cancer cachexia [40]. Hence, 3T3L1 cells were used to analyze the protein expression level of ZAG, HSL, and p-HSL (563 and 565) by Western blot analysis. As shown by the results (Figure 4C), tumor-derived CM can induce the expression of ZAG and p-HSL. However, BGM treatment substantially decreased the ZAG and p-HSL (563 and 565) levels in both BxPC-3 and MIA PaCa-2 CM.

### 2.7. BGM Inhibits Cachexia-Induced Inflammatory Signaling Factors in 3T3L1 Adipocytes In Vitro

To confirm the optimal time point at which various tumorigenesis factors can display the maximal activity in 3T3L1 adipocytes, cells were treated with BGM at various time intervals (Figure 4D). We noted that both CM of BxPC-3 and MIA PaCa-2 induced the maximal STAT3 and ERK phosphorylation at the 15 min time point. Thereafter, the impact of BGM on STAT3 and ERK activation in 3T3L1 cells was deciphered (Figure 4E). The data suggested that CM induced STAT3 and ERK phosphorylation whereas BGM suppressed the expression of various proteins with increasing concentrations.

### 2.8. BGM Suppresses Cancer Cachexia and Modulates the Expression of Skeletal Muscle- and Adipogenesis-Related Factors In Vivo in a Xenograft Mouse Model

**Figure 5:**
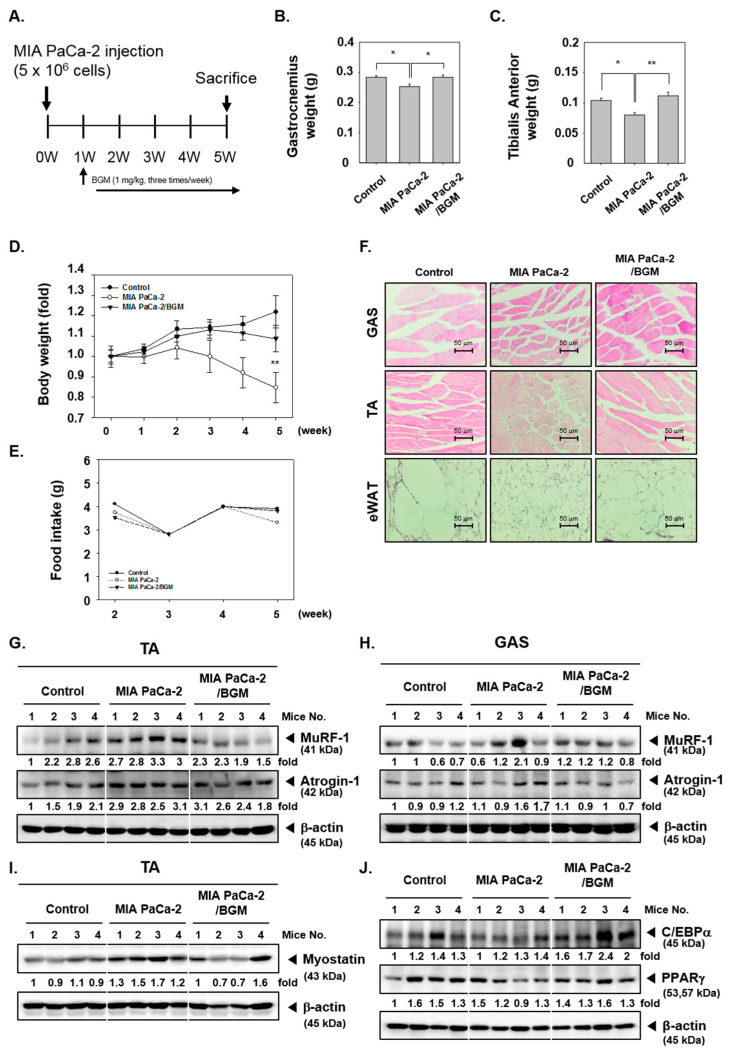
The suppression of muscle and adipocyte tissues atrophy in animal models upon BGM exposure. (**A**) MIA PaCa-2 cells (1 × 10^6^ cells/mice) were injected subcutaneously into the right flank of the mice. After 1 week, the animals were randomized into 3 groups. Group I consisted of control mice, group II mice were injected MIA PaCa-2 cells, and group III mice were exposed to BGM (1 mg/kg) after MIA PaCa-2 cells injection. The mice were treated 3 times a week with BGM for 4 weeks and then sacrificed after 5 weeks. (**B**,**C**) Gastrocnemius (GAS) and tibialis anterior (TA) weight was measured on the last day of the experiment (mean ± SE). (**D**,**E**) Five-week change of body weight and food intake. (**F**) GAS, TA, and epididymal white adipose tissue (eWAT) were obtained from each control, MIA PaCa-2, and MIA PaCa-2/BGM groups were stained to compare cancer cachexia-induced atrophy. Scale bar = 50 μm. (**G**,**H**) The expression levels of E3 ubiquitin ligases *Atrogin-1* and *MuRF-1* were analyzed on TA and GAS by Western blot analysis. For this purpose, each sample was obtained from individual mice in an individual group. (**I**) Myostatin expression in TA was analyzed by Western blot analysis. (**J**) C/EBPα and PPARγ expression levels in eWAT were observed by Western blot analysis. All the experiments were performed independently at least 3 times, and representative data are shown. Data represent means ± SD. * *p* < 0.05 vs. BGM, ** *p* < 0.01 vs. Control.

We examined the effects of BGM in MIA PaCa-2, human pancreatic cancer cells, and xenograft mouse. MIA PaCa-2 xenograft was developed to induce cancer cachexia in mice. The experiments were conducted as per the schedule depicted in Figure 5A. After sacrifice, gastrocnemius (GAS) and tibialis anterior (TA) were obtained, and weight was measured for comparison (Figure 5B,C). In Figure 5D,E, cachexia-induced mice displayed a significant loss of the body weight; however, BGM can significantly recover the weight loss. On the other hand, there was no significant change on food intake for five consecutive weeks. GAS and TA weights of the MIA PaCa-2 xenograft group were noted to be decreased as compared to those of the control group, but the weight losses were suppressed in the BGM-treated group. Thereafter, GAS, TA, and epididymal white adipose tissue (eWAT) were Hematoxylin and Eosin (H&E)-stained to evaluate cancer cachexia-induced atrophy (Figure 5F). In the MIA PaCa-2 xenograft group, cachexia was observed with the atrophy of the GAS, TA, and eWAT. However, atrophy was significantly suppressed upon the BGM treatment. To evaluate the skeletal muscle- and adipogenesis-related factors expression in the xenograft mouse model, isolated tissues were analyzed by Western blot analysis. Muscle atrophy-related factors *MuRF-1* and *Atrogin-1* levels were analyzed in TA and GAS (Figure 5G,H). Interestingly, the expression levels of *MuRF-1* and *Atrogin-1* were induced in the cachexia group, but BGM downregulated the levels of these proteins. In addition, myostatin, which can inhibit myogenesis, was induced in the cachexia group and suppressed upon BGM exposure (Figure 5I). Finally, the levels of adipogenesis regulation factors C/EBPα and PPARγ were evaluated with eWAT (Figure 5J). It was found that both C/EBPα and PPARγ levels were inhibited by cachexia, but BGM recovered and substantially increased the expression levels of these two proteins.

## 3. Discussion

The goal of this study was to determine the modulatory actions of BGM on cancer cachexia and decipher its underlying molecular mechanism. Although cachexia is a multifactorial disease, in this study we primarily focused on cachexia caused by cancer and investigated the mechanisms leading to its development [22,41,42] and how various events-mediating cachexia can be affected by BGM. As the typically reported symptoms of cancer cachexia include weight loss, anorexia, asthenia, and anemia, we investigated the impact of BGM signaling pathways related to muscle and fat atrophy [1,2,3,4].

Muscle atrophy has been established as one of the major symptoms of cachexia [6,42]. There are various factors that induce muscle loss, including *Atrogin-1* and *MuRF-1* that can act as representative of the muscle atrophy process. For instance, Bodine et al. reported that *Atrogin-1* and *MuRF-1* expression levels were increased in resting skeleton or under protein catabolic conditions. *Atrogin-1* and *MuRF-1* are E3 ubiquitin ligases and have been used as cachexia markers, because they play a key role in regulating muscle atrophy [10,11,43]. We induced cancerous cachexia in C2C12 cells using pancreatic cells CM, established the morphological changes characteristics of cachexia and observed an increase in the expression levels of Atrogin-1 and MuRF-1 proteins. Under these conditions, the effect of inhibiting muscle atrophy was demonstrated through the suppression of Atrogin-1 and MuRF-1 by exposing the cells to BGM. We noticed that cancer cachexia inhibited the expression of MyHC, a skeletal muscle gene products, and it was observed that BGM effectively restored the MyHC expression [39,44]. Moreover, cancer cachexia increased the levels of autophagy activity and tumorigenesis factors in muscle cells, but BGM showed a marked inhibitory effect against both the phenomena [45,46,47,48]. These results suggested that the cancer cachexia inhibitory effect of BGM can predominantly function by inhibiting muscle atrophy and tumorigenesis while increasing the expression of musculoskeletal factors such as MyHC.

Another typical symptom commonly associated with cancerous cachexia is adipocyte atrophy. Adipocyte atrophy can lead to a reduction of cell volume and decrease de novo lipogenesis [14,15,16,17]. Adipocytes and adipose tissues can play an important role in this process by controlling hormone regulation and inflammatory mediators production in the body [18]. We treated pancreatic cancer cell CM to observe adipocyte atrophy due to cancer cachexia in 3T3L1 cells and observed a dramatic decrease in the process of adipogenesis by oil red O staining. C/EBPα and PPARγ can act as adipogenesis regulation factors, and aP2, adiponectin, and resistin can control adipocyte specific gene expression [20,21]. Cancer cachexia development can cause a downregulation of these factors; however, BGM prevented the suppression of adipocyte differentiation by increasing adipogenesis factors and the levels of related hormone. It was also confirmed that BGM suppressed cancer cachexia-induced inflammatory signaling in adipocyte, which indicates the versatility of this furanocoumarin to attenuate the progression of cancer and symptoms associated with this dreaded disease.

We also investigated cancer cachexia progression in animal model muscles and adipose tissues. A xenograft model with MIA PaCa-2 human pancreatic cancer cells in mice was used to induce cancer cachexia, and significant weight losses were observed in the representative muscle tissues GAS and TA. In addition, muscle atrophy was observed by H&E staining, and increased levels of both MuFR-1 and Atrogin-1 were noted. On the contrary, in the mice treated with BGM, both the weight loss of muscle tissue and atrophy were substantially suppressed. The Western blot results with muscle tissues further demonstrated that BGM can effectively diminish MuFR-1 and Atrogin-1 expression while increasing the levels of Myostatin. The adipose tissue eWAT could also inhibit adipogenesis and the expression of adipose differentiation factors in cachexia-induced mice, but it was found that adipocyte atrophy was dramatically restored in BGM-treated mice. Thus, these results stablished that inhibitory actions of BGM on cancer cachexia were clearly effective under both in vitro and in vivo conditions.

## 4. Materials and Methods

### 4.1. Reagents

BGM was purchased from Sigma-Aldrich (St. Louis, MO, USA). In addition, 3-(4,5-dimethylthiazol-2-yl)-2,5-diphenyltetrazolium bromide (MTT), Tris base, glycine, NaCl, sodium dodecylsulfate (SDS), oil red O solution, and autofluorescent agent monodansylcadaverine (MDC) were purchased from Sigma-Aldrich (St. Louis, MO, USA). Anti-atrogin-1, anti-MuRF-1, anti -STAT3, anti-akt, anti-C/EBPα, anti-PPARγ, anti-resistin, anti-myostatin, and anti-β-actin antibodies were purchased from Santa Cruz Biotechnology (Santa Cruz, CA, USA). Anti-phospho-STAT3, anti-phospho-Akt, anti-phospho-FoxO4, anti-FoxO4, anto-aP2, anti-adiponectin, anti-phospho-ERK, and anti-ERK antibodies were purchased from Cell Signaling Technology (Beverly, MA, USA).

### 4.2. Cell Lines and Culture Conditions

Mouse myoblast C2C12 and mouse embryo fibroblast cells were obtained from American Type Culture Collection (Manassas, VA, USA). C2C12 cells were cultured in DMEM/high medium containing 10% fetal bovine serum (FBS). When inducing C2C12 cells differentiation, cells were incubated with DMEM/high medium containing 2% horse serum for 3 days. 3T3-L1 cells were cultured in DMEM medium containing 10% FBS and 1% PSG. To induce the cell differentiation, cells were incubated with MDI media (IBMX, Dexamethasone, Insulin) for 3 days. Cells conditions were maintained at 37 °C in 5% CO_2_ conditions.

### 4.3. MTT Assay

C2C12 cells were seeded incubated until the density reaches 90% with DMEM/high medium containing 10% FBS, then change medium to differentiation by DMEM/high medium containing 2% horse serum for 3 days. After differentiation, human pancreas cancer BxPC-3 and MIA PaCa-2 cells CM were treated with BGM for 48 h. Then cell viability was measured as described previously [49,50].

### 4.4. Western Blot Analysis

Western blot analysis was performed as described previously [51,52]. Detailed information about Western Blot can be found at Supplementary Materials.

### 4.5. MDC Staining

MDC staining was examined to evaluate the autophagy activation in 3T3-L1 cells. After treatment, cells were washed by 1× PBS and then stained with 50 µM MDC at 37 °C for 10 min. The cells were analyzed by BD Accuri™ C6 Plus Flow Cytometer (BD Biosciences, Becton-Dickinson, Franklin Lakes, NJ, USA) with BD Accuri C6 Plus software (BD Accuri C6 Plus, BD Biosciences, Franklin Lakes, NJ, USA).

### 4.6. Oil Red O Staining

3T3-L1 cells were seeded and incubated until the density reached 90% with a DMEM medium containing 10% FBS and 1% PSG, and then the medium was replaced with MDI media (IBMX, dexamethasone, and insulin) for 2 days. After differentiation to adipocyte, the cells were treated with insulin (1 µg/mL) containing BxPC-3 and MIA PaCa-2 cells CM with BGM for 2 days. After treatment, the cells were fixed by 10% formalin for 1 h, washed by 60% isopropanol and then dried at room temperature. After drying, the cells were stained by 60% oil red O solution for 1 h and washed with distilled water. The cells were observed using by Nikon ECLIPSE Ts2 (Nikon, Tokyo, Japan, magnification: 20×). Thereafter, the cells were lysed with 60% isopropanol and measured by VARIOSKAN LUX (Thermo Fisher Scientific Inc, Waltham, MA, USA) at 550 nm.

### 4.7. Animals

All procedures involving animals were reviewed and approved by the Kyung Hee University Institutional Animal Care and Use committee [KHUASP(SE)-18-170] (approval date: 19 August 2016). Six-week-old athymic nu/nu male mice were purchased form Nara Biotec CO. (Gyeonggi-do, Korea).

### 4.8. Experimental Protocol

One week after tumor injection, tumor diameters were measured using the Digimatic caliper (Mitutoyo Company, Kawasaki, Japan). When tumors reached 0.25 cm in diameter, the mice were randomized into 3 treatment groups (*n* = 6/group): Group I as the control group, group II comprised of mice injected with MIA PaCa-2 cells (5 × 10^6^ cells/mice), and group III consisting of mice exposed to BGM (1 mg/kg; intraperitoneal (i.p.) injection: 3 times a week) after MIA PaCa-2 cells injection. The Therapy was continued for 4 weeks from the randomization (0 week). Mice were killed 5 days later form last therapy. GAS, TA, and epididymal white adipose tissue (eWAT) were excised, and the final weight as well as the volume was measured using the formula: V = 4/3 πr^3^. Half of the tumor tissues were fixed in formalin and embedded in paraffin for immunohistochemistry and routine H&E staining. The other half was snap frozen in liquid nitrogen and stored at −80 °C.

### 4.9. Western Blot Analysis of Tumor Tissues

Western blot analysis was performed as described previously [51]. Detailed information about Western Blot can be found at Supplementary Materials.

### 4.10. H&E Staining

GAS, TA, and eWAT were processed and embedded in paraffin. The sections were cut and deparaffinized in xylene, dehydrated in graded alcohol and finally hydrated in water. The sections were stained with hematoxylin for 3 min, washed, reacted with 1% HCL and alcohol for 10 s, and then incubated with 0.5% ammonia water for 1 min. After a 20 s reaction with eosin, the dehydration was carried out and the sections were mounted. The samples were observed by Nikon ECLIPSE Ts2 (magnification: 20×).

### 4.11. Statistical Analysis

The results were expressed as means ± SD, and an analysis of variance (ANOVA) with Bonferroni’s test was used for the statistical analysis of multiple comparisons of data. *p*-value of 0.05 or less was considered as significant.

## 5. Conclusions

Overall, this study established the potential impact of BGM on the attenuation of cancer cachexia that can significantly improve the quality of life of patients by assisting in the treatment options available for cancer cachexia. Furthermore, BGM appears to function as a novel therapeutic agent that can be effectively employed for the treatment of various cancer-related complications to improve the overall survival. However, further studies are needed to understand the exact mechanism of the action of BGM in affecting cancer cachexia.

## Data Availability

The data presented in this study are available on request from the corresponding author.

## Supplementary Materials

The following are available online at https://www.mdpi.com/2072-6694/13/6/1347/s1, detailed information about Western Blot.

## Abbreviations

BGM	Bergamottin
STAT3	signal transducer and activator of transcription 3
c/w	cell per well
FBS	fetal bovine serum
HRP	horseradish peroxidase
H&E	Hematoxylin and Eosin
i.p.	intraperitoneal
CM	Conditioned media
MyHC	myosin heavy chain
NT	nontreated
P/S	penicillin–streptomycin
ZAG	Zinc-α2 glycoprotein
HSL	hormone-sensitive lipase
SFM	serum-free media
TA	Tibialis anterior
GAS	gastrocnemius
eWAT	epididymal white adipose tissue
