# Supplementary material for: A Novel Role of Bergamottin in Attenuating Cancer Associated Cachexia by Diverse Molecular Mechanisms
Source: Cancers (Basel). 2021 Mar 17;13(6):1347. doi: 10.3390/cancers13061347 (PMC8002497; doi:10.3390/cancers13061347)
Supplement: Supplementary file 1 [file cancers-13-01347-s001.pdf]

Supplementary Materials

A Novel Role of Bergamottin in Attenuating Cancer Associated Cachexia by Diverse Molecular Mechanisms

Young Yun Jung, Jeong-Hyeon Ko, Jae-Young Um, Gautam Sethi and Kwang Seok Ahn

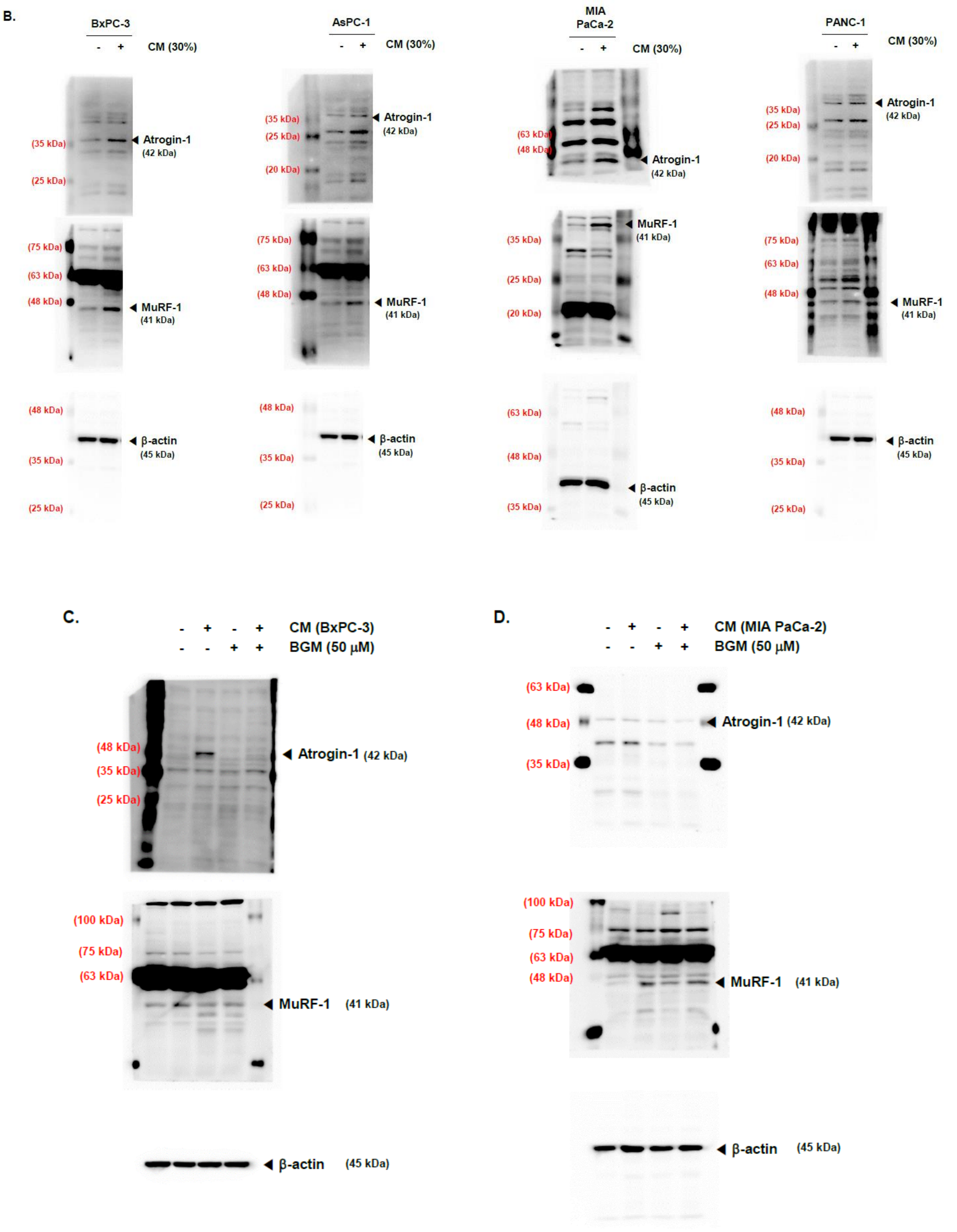

Detail information about Figure 1.

A.

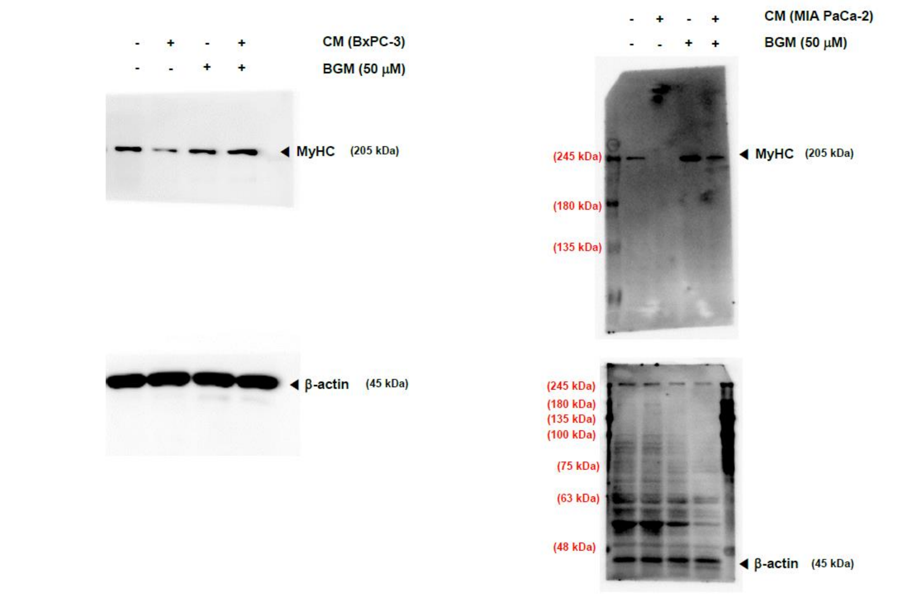

- + - + CM (BxPC-3)
- - + + BGM (50 μM)
MyHC (205 kDa)
β-actin (45 kDa)
- + - + CM (MIA PaCa-2)
- - + + BGM (50 μM)
(245 kDa)
(180 kDa)
(135 kDa)
MyHC (205 kDa)
(245 kDa)
(180 kDa)
(135 kDa)
(100 kDa)
(75 kDa)
(63 kDa)
(48 kDa)
β-actin (45 kDa)

B.

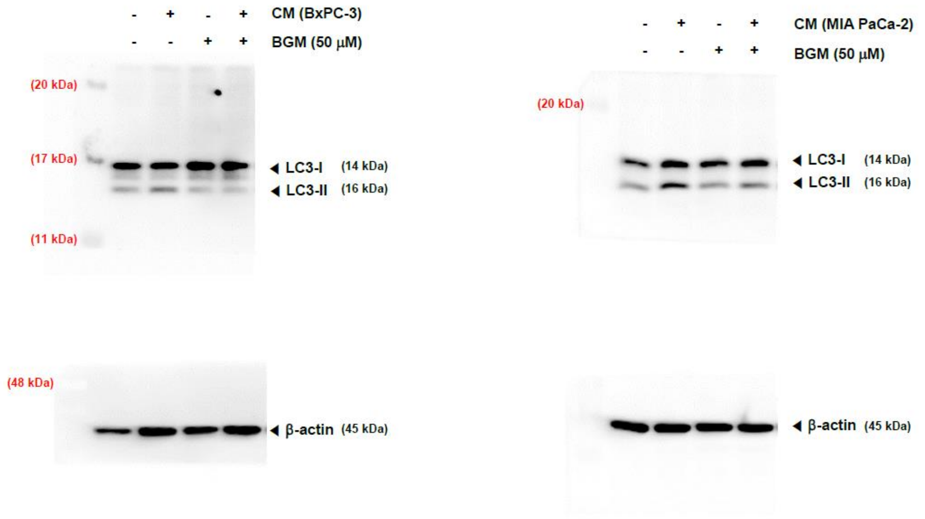

- + - + CM (BxPC-3)
- - + + BGM (50 μM)
(20 kDa)
(17 kDa)
(11 kDa)
LC3-I (14 kDa)
LC3-II (16 kDa)
(48 kDa)
β-actin (45 kDa)
- + - + CM (MIA PaCa-2)
- - + + BGM (50 μM)
(20 kDa)
LC3-I (14 kDa)
LC3-II (16 kDa)
β-actin (45 kDa)

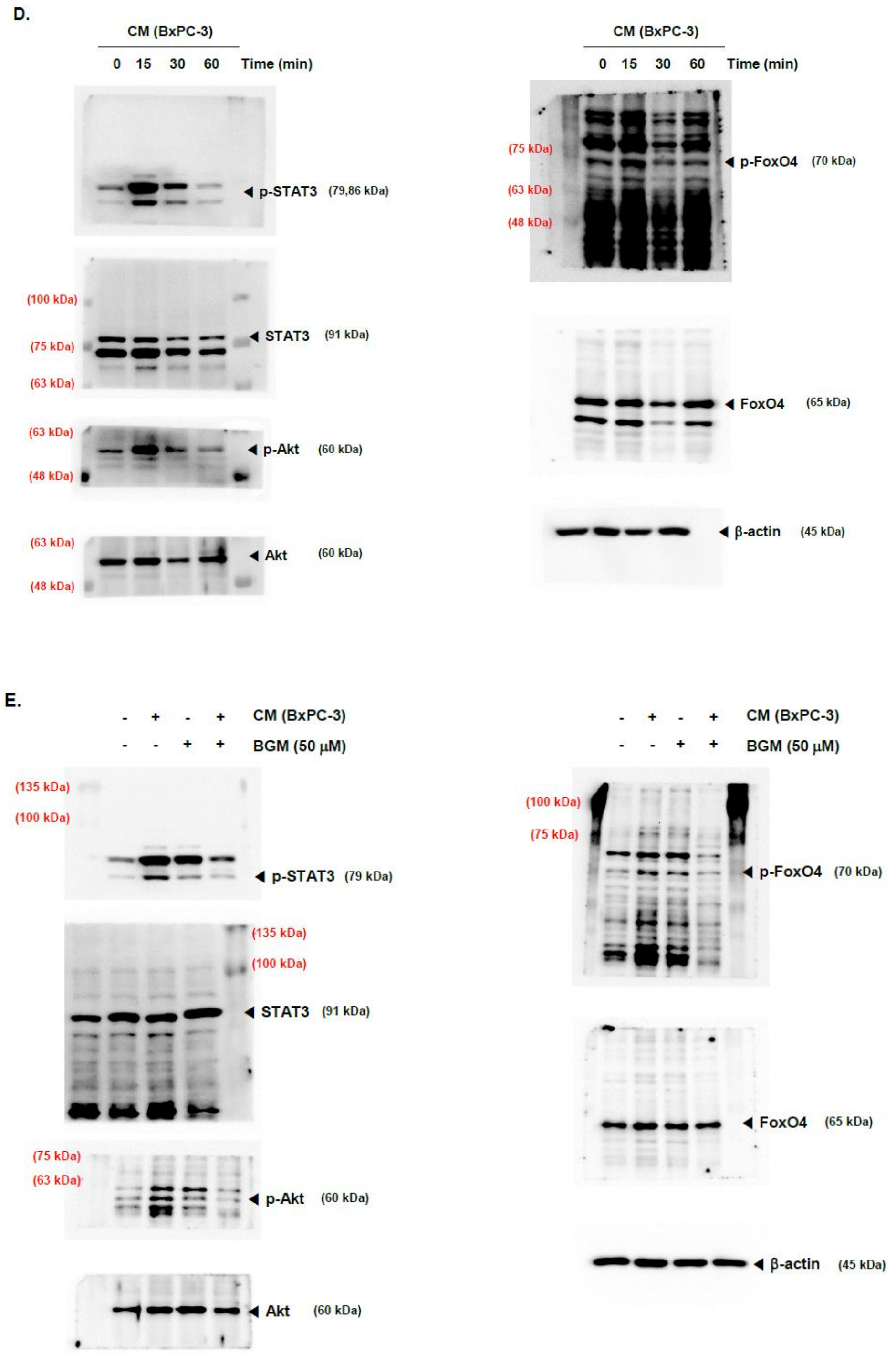

Detail information about Figure 2.

A.

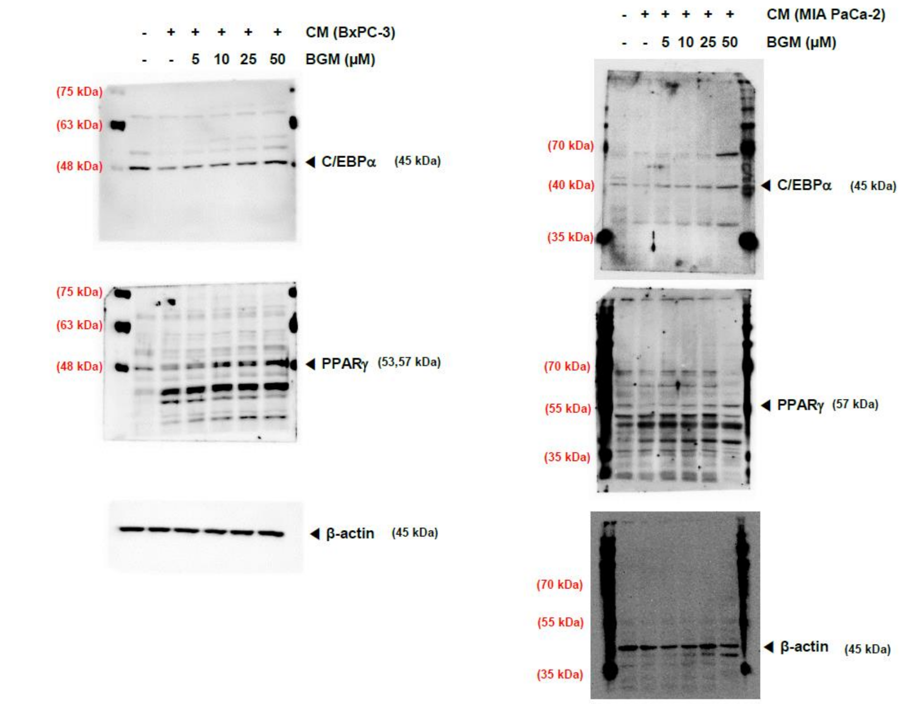

CM (BxPC-3)
BGM (μM)
- + + + + +
- - 5 10 25 50
(75 kDa)
(63 kDa)
(48 kDa)
C/EBPα (45 kDa)
PPARγ (53,57 kDa)
β-actin (45 kDa)
CM (MIA PaCa-2)
BGM (μM)
(70 kDa)
(40 kDa)
(35 kDa)
C/EBPα (45 kDa)
(55 kDa)
PPARγ (57 kDa)
β-actin (45 kDa)

B.

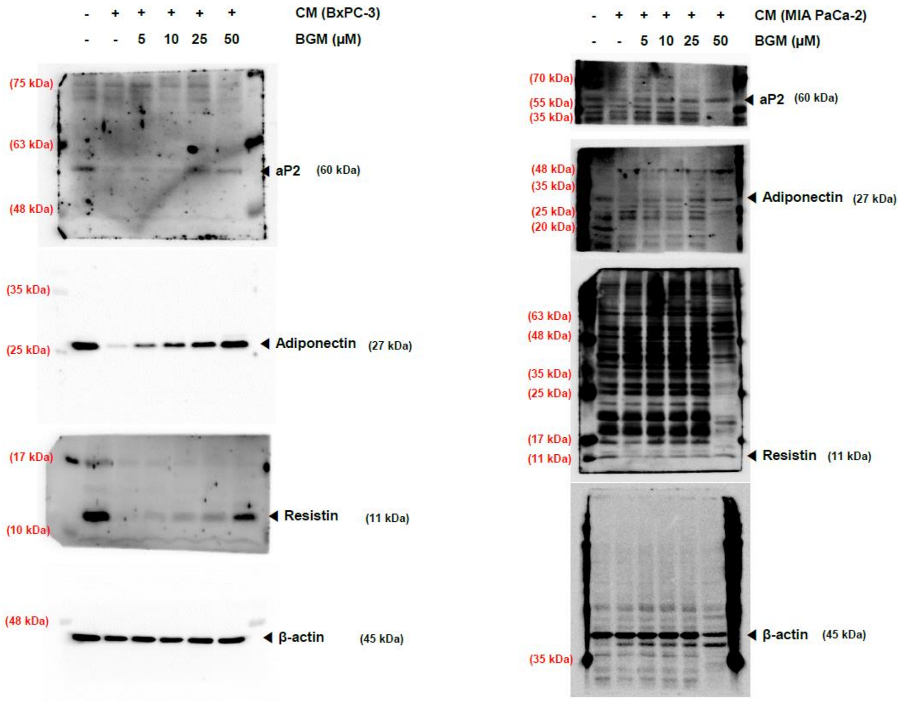

CM (BxPC-3)
BGM (μM)
- + + + + +
- - 5 10 25 50
(75 kDa)
(63 kDa)
(48 kDa)
aP2 (60 kDa)
(35 kDa)
(25 kDa)
Adiponectin (27 kDa)
(17 kDa)
(10 kDa)
Resistin (11 kDa)
β-actin (45 kDa)
CM (MIA PaCa-2)
BGM (μM)
(70 kDa)
(55 kDa)
aP2 (60 kDa)
(20 kDa)
Adiponectin (27 kDa)
(11 kDa)
Resistin (11 kDa)
β-actin (45 kDa)

C.

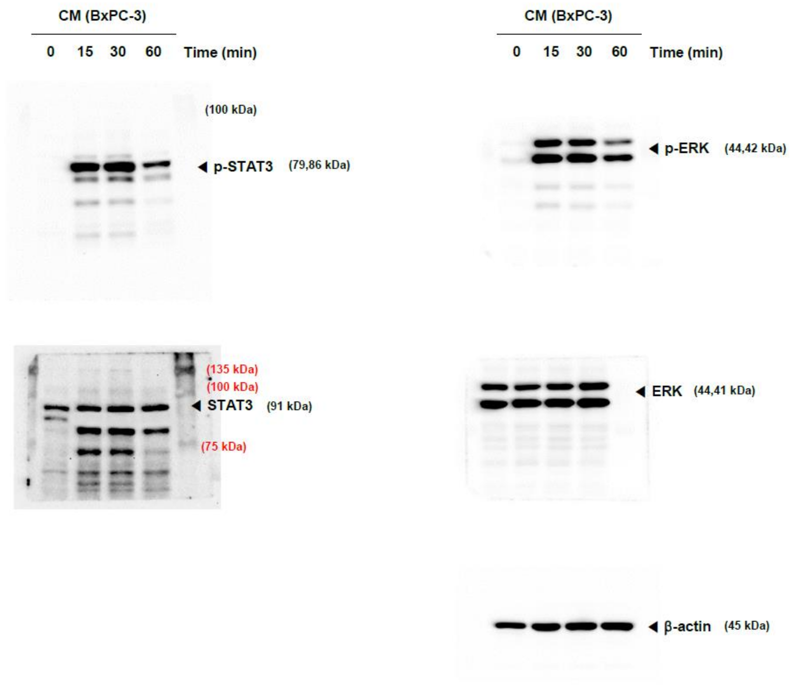
CM (BxPC-3)
0 15 30 60 Time (min)
(100 kDa)
p-STAT3 (79,86 kDa)
(135 kDa)
(100 kDa)
STAT3 (91 kDa)
(75 kDa)
CM (BxPC-3)
0 15 30 60 Time (min)
p-ERK (44,42 kDa)
ERK (44,41 kDa)
β-actin (45 kDa)

C.

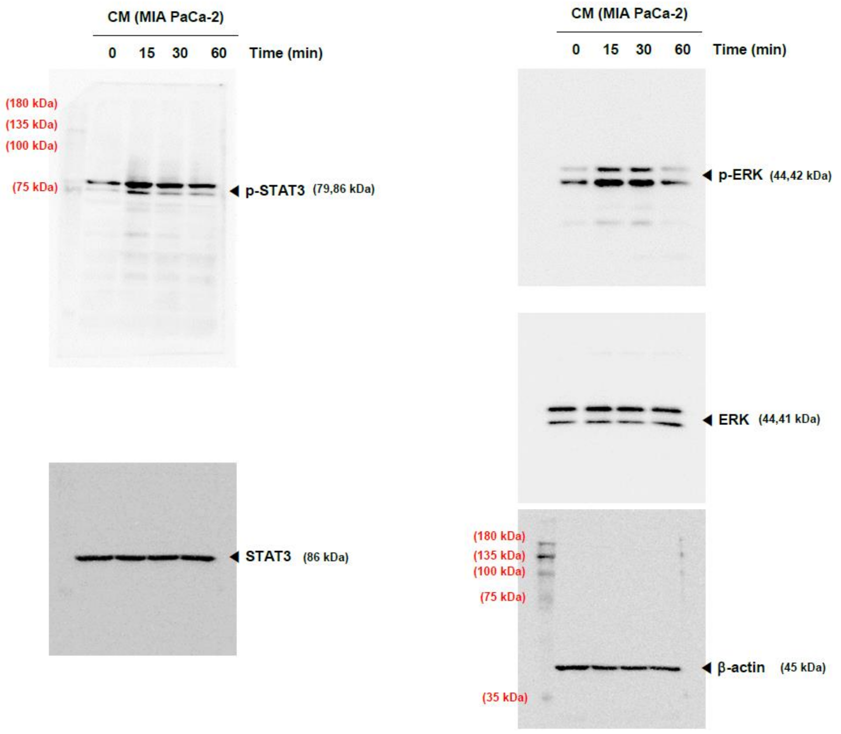
CM (MIA PaCa-2)
0 15 30 60 Time (min)
(180 kDa)
(135 kDa)
(100 kDa)
(75 kDa)
p-STAT3 (79,86 kDa)
STAT3 (86 kDa)
CM (MIA PaCa-2)
0 15 30 60 Time (min)
p-ERK (44,42 kDa)
ERK (44,41 kDa)
(180 kDa)
(135 kDa)
(100 kDa)
(75 kDa)
β-actin (45 kDa)
(35 kDa)

D.

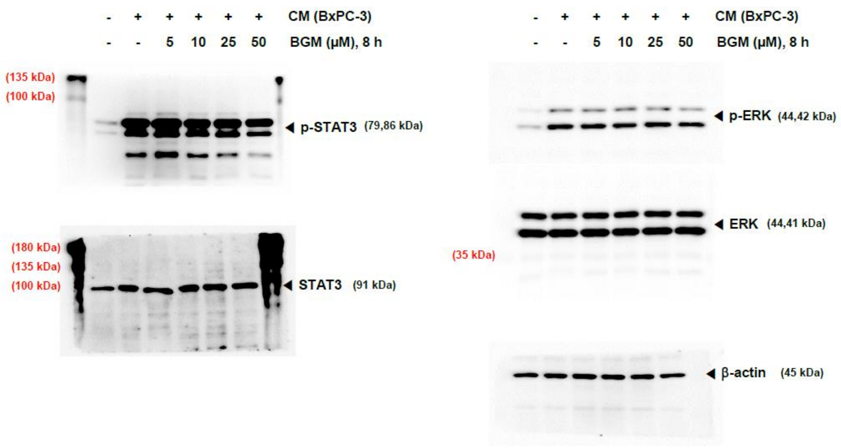

D.

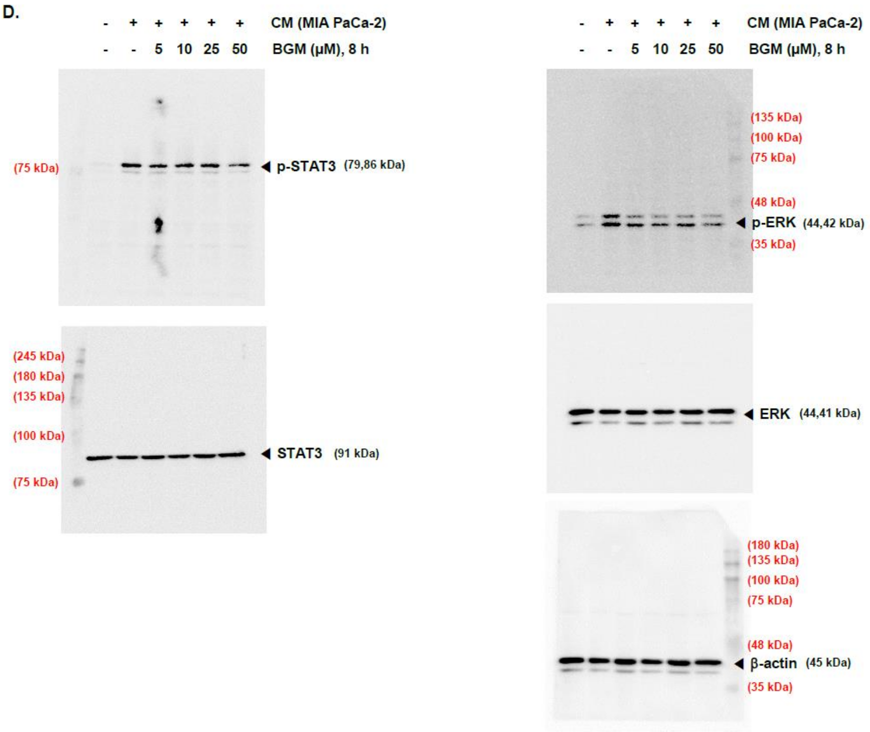

Detail information about Figure 4.

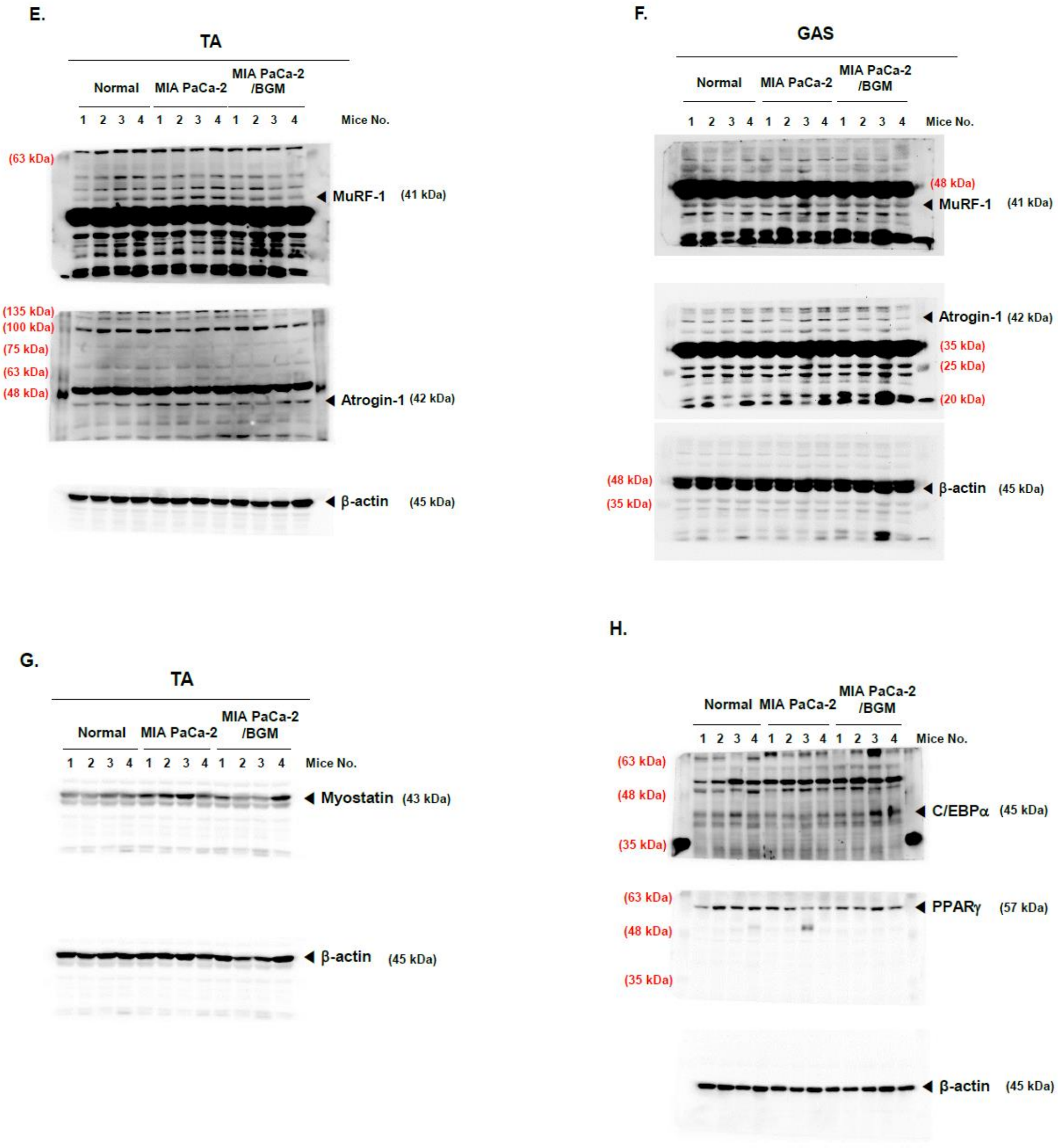

Detail information about Figure 5.

© 2021 by the authors. Licensee MDPI, Basel, Switzerland. This article is an open access article distributed under the terms and conditions of the Creative Commons Attribution (CC BY) license (http://creativecommons.org/licenses/by/4.0/).
